# Pinpointing Brain TREM2 Levels in Two Mouse Models of Alzheimer’s Disease

**DOI:** 10.1007/s11307-021-01591-3

**Published:** 2021-02-23

**Authors:** Silvio R. Meier, Dag Sehlin, Greta Hultqvist, Stina Syvänen

**Affiliations:** 1 Department of Public Health and Caring Science/Molecular Geriatrics, Rudbecklaboratoriet, Uppsala University, Dag Hammarskjölds Väg 20, Uppsala, Sweden; 2 Department of Pharmaceutical Biosciences/Protein Drug Design, BMC, Uppsala University, Husargatan 3, Uppsala, Sweden

**Keywords:** Alzheimer’s disease, PET, TREM2, sTREM2, Bispecific antibody, Neuroinflammation

## Abstract

**Purpose:**

The triggering receptor expressed on myeloid cells 2 (TREM2) is expressed by brain microglia. Microglial activation, as observed in Alzheimer’s disease (AD) as well as in transgenic mice expressing human amyloid-beta, appears to increase soluble TREM2 (sTREM2) levels in CSF and brain. In this study, we used two different transgenic mouse models of AD pathology and investigated the potential of TREM2 to serve as an *in vivo* biomarker for microglial activation in AD.

**Procedures:**

We designed and generated a bispecific antibody based on the TREM2-specific monoclonal antibody mAb1729, fused to a single-chain variable fragment of the transferrin receptor binding antibody 8D3. The 8D3-moiety enabled transcytosis of the whole bispecific antibody across the blood-brain barrier. The bispecific antibody was radiolabeled with I-125 (*ex vivo*) or I-124 (PET) and administered to transgenic AD and wild-type (WT) control mice. Radioligand retention in the brain of transgenic animals was compared to WT mice by isolation of brain tissue at 24 h or 72 h, or with *in vivo* PET at 24 h, 48 h, and 72 h. Intrabrain distribution of radiolabeled mAb1729-scFv8D3_CL_ was further studied by autoradiography, while ELISA was used to determine TREM2 brain concentrations.

**Results:**

Transgenic animals displayed higher total exposure, calculated as the AUC based on SUV determined at 24h, 48h, and 72h post injection, of PET radioligand [^124^I]mAb1729-scFv8D3_CL_ than WT mice. However, differences were not evident in single time point PET images or SUVs. *Ex vivo* autoradiography confirmed higher radioligand concentrations in cortex and thalamus in transgenic mice compared to WT, and TREM2 levels in brain homogenates were considerably higher in transgenic mice compared to WT.

**Conclusion:**

Antibody-based radioligands, engineered to enter the brain, may serve as PET radioligands to follow changes of TREM2 *in vivo*, but antibody formats with faster systemic clearance to increase the specific signal in relation to that from blood in combination with antibodies showing higher affinity for TREM2 must be developed to further progress this technique for *in vivo* use.

**Supplementary Information:**

The online version contains supplementary material available at 10.1007/s11307-021-01591-3.

## Introduction

Alzheimer’s disease (AD) is the most common form of dementia. The number of people suffering from AD is expected to increase, mainly due to an older population worldwide as age is the key risk factor for the disease [1]. The complex mechanisms of the disease are not fully understood. Besides the classical disease hallmarks such as extracellular aggregation of the protein amyloid-beta (Aβ) and the formation of intracellular neurofibrillary tau-tangles, glial cells respond strongly to these pathological changes. The resulting inflammatory reaction is believed to be directly linked to the pathogenesis of the disease and may contribute to disease progression and severity [2]. Microglia are resident immune cells of the brain that constantly monitor the environment for signals related to damage [3]. Microglial cells express a wide range of immune receptors. Several missense mutations of the triggering receptor expressed on myeloid cells 2 (TREM2) have previously been reported to increase the risk of developing AD [4]. The receptor undergoes intramembrane proteolysis [5] leading to the release of the extracellular soluble part of TREM2 (sTREM2). sTREM2 can be detected in CSF, and concentrations are already increased in early symptomatic stages of AD [6]. In addition, sTREM2 concentrations appear to be correlated with markers for neurodegeneration, e.g., CSF concentrations of total tau and phosphorylated-tau [7, 8]. Brendel et al. (2017) studied sTREM2 levels in brain homogenates obtained from PS2APP mice (transgenic AD model) and reported an age-dependent increase in transgenic mice compared with wild-type (WT) mice [9]. The strong correlation between total Aβ and sTREM2 reported by Brendel et al. supports the association between amyloidosis and increased sTREM2 [10]. Further, *post mortem* analysis of sTREM2 concentrations in brain homogenates was compared to positron emission tomography (PET) imaging using the translocator protein (TSPO) radioligand (4*S*)-*N*,*N*-diethyl-9-(2-(^18^F)fluoranylethyl)-5-methoxy-1,2,3,4-tetrahydrocarbazole-4-carboxamide ([^18^F]GE180), which images microglial activation, and 4-[(E)-2-(4-{2-[2-(2-[^18^F]fluoroethoxy)ethoxy]ethoxy}phenyl)vinyl]-*N*-methylaniline ([^18^F]florbetaben), which is used to quantify brain Aβ plaque load [10]. *In vivo*, TSPO levels correlated well with both sTREM2 and amyloid levels. Interestingly, microglial activation, measured with [^18^F]GE180, was reported already at an early age when Aβ deposition was sparse and only detectable by sensitive histological methods but not with [^18^F]florbetaben PET. This indicates that sTREM2 might serve as a biomarker for microglial activation at a very early disease stage, and thus, it would be beneficial to image and quantify sTREM2 concentrations *in vivo* with PET imaging. This approach could be an alternative to TSPO PET radioligands that suffer from many challenges such low brain distribution, unspecific and genotype-dependent binding.

Antibodies and antibody fragments have recently been described as PET radioligands for different aggregation forms of Aβ in rodents [11–16]. To cross the blood-brain barrier (BBB), the antibodies, which are too large for passive brain distribution, were fused to a transferrin receptor (TfR) binding moiety based on the 8D3 antibody [17, 18] that enabled active transport across the BBB into cerebral tissue. This strategy increased the brain distribution of the antibodies up to 80-fold, improved the spatial distribution inside the brain parenchyma, and allowed for their use, after radiolabeling, as *in vivo* PET radioligands [14, 19, 20]. In the brain, these constructs profit from a very selective and strong binding to the target structure, i.e., Aβ in the previously mentioned studies.

The aim of this study was to investigate TREM2 levels in two transgenic AD mouse models and WT mice and the potential age-dependent changes. Further, we aimed to create a bispecific radioligand that would enable *in vivo* visualization and quantification of TREM2 with PET. *Ex vivo* measurement of TREM2 in brain homogenates is likely to correspond to sTREM2 rather than to membrane bound TREM2. However, it should be noted that *in vivo*, the developed bispecific radioligand could also bind to membrane bound TREM2. Therefore, we have decided to use “TREM2” throughout the manuscript, except when explicitly referring to ELISA measurements in TBS brain extracts.

## Materials and Methods

### Bispecific Antibody Preparation

The bispecific antibody was based on the anti-TREM2 antibody mAb1729 (R&D, Abingdon, UK) and a modified single-chain variable fragment (scFv) of the TfR binding antibody 8D3 [18] optimized for cross-linking, scFv8D3_CL_. The scFv8D3_CL_ was generated by adding a lysine-rich sequence at the C-terminus with double FLAG-Tag [21] (2xDYKDDDK) to promote NHS-ester binding at the C-terminus of the scFv8D3. A linker was placed between the scFv and the lysine-rich part. The linker consisted of amino acid 1–7 of the sequence of mouse Ig lambda-1 C_L_ (QPKSSPS) as an endogenous extension of a scFv at its C-terminus. The linker increases the distance and thus flexibility of the added moiety to promote accessibility for subsequent trans-cyclooctene (TCO) functionalization. For nickel purification, a 6xHis tag was added at the very end of the C-terminus. The expression of scFv8D3_CL_ was performed as described by Fang et al. (2018) [22].

**Fig. 1:**
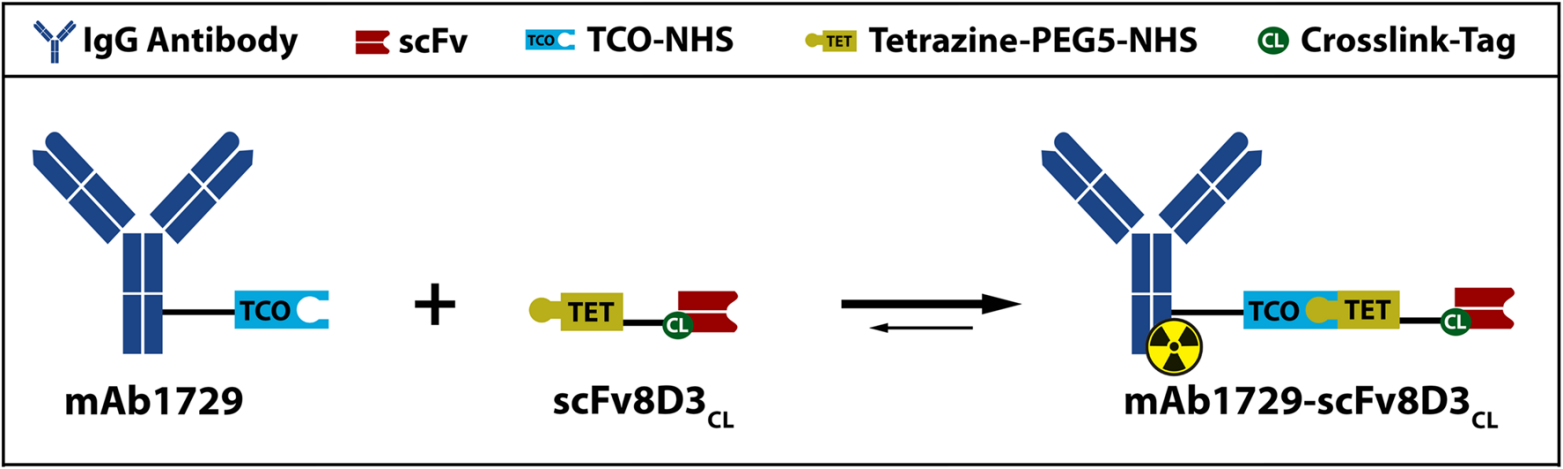
mAb1729 and scFv8D3_CL_ were linked to TCO and tetrazine groups, respectively, by NHS-ester conjugation. The functionalized proteins were then conjugated by Diels-Alder reaction to generate the bispecific protein mAb1729-scFv8D3_CL_, with the ability to bind both TREM2 and the transferrin receptor. The bispecific protein was radiolabeled with I-125 or I-124 for *ex vivo* and PET studies, respectively.

In house expressed scFv8D3_CL_ was chemically conjugated to mAb1729 using TCO-tetrazine Diels-Alder “click chemistry“ [23] (Fig. 1). Antibody mAb1729 (R&D), 250 μg (2 mg/ml), was incubated for 2 h with a 7-fold excess of axial TCO-NHS (Conju-probe, San Diego, USA) resulting in TCO-conjugation to the antibody. The scFv8D3_CL_ (2 mg/ml) was incubated with a 5-fold excess of tetrazine-PEG5-NHS (Sigma Aldrich, Stockholm, Sweden). Both reactions were stopped by running the reaction solution over desalting columns Zeba 7 kDa (Fisher scientific, Gothenburg, Sweden). Modified proteins were mixed with 10-fold molar excess of scFv8D3_CL_ and incubated at room temperature for 5 h. Proteins were separated by gel filtration using an ÄKTA Start system (GE Healthcare, Uppsala, Sweden) with a HiPrep 16/60 Sephacryl S-100 column (Sigma). The final preparation resulted in 79 % single or multiple conjugated mAb1729-scFv8D3_CL_, 3 % unconjugated scFv8D3_CL_, and 18 % unconjugated mAb1729.

### Radiolabeling and Binding ELISA

Bispecific antibody mAb1729-scFv8D3_CL_ was radiolabeled with I-125 or I-124 (PerkinElmer Inc., Waltham, MA, USA) by direct radioiodination [24]. I-125 labeling: 35 μg of protein was diluted in PBS to a concentration of 0.4 mg/ml. 24.5±5 MBq of I-125 was added followed by 5 μl chloramine T (1 mg/ml). After 90-s incubation, the reaction was quenched with 10 μl of sodium metabisulfite (1 mg/ml). The volume was extended to 0.5 ml with PBS and transferred to a NAP-5 size-exclusion column (GE Healthcare). The labeled protein was eluted with 1 ml PBS collected in 2 fractions (900 μl, 100 μl). Only the first fraction was used for injections. I-124 labeling: 65 μg of protein was diluted in PBS to a concentration of 0.2 mg/ml. 130±4MBq I-124 was added immediately followed by 40 μl chloramine T (1 mg/ml) and incubated for 120 s. The reaction was quenched by adding 80 μl of sodium metabisulfite (1 mg/ml). The protein was separated from free iodine as described above. Protein labeling yields were 45–50 % for I-125 and 65–70 % for I-124. Binding of labeled and unlabeled mAb1729-scFv8D3_CL_ was investigated with ELISA to ensure that the affinity to TREM2 and TfR was intact also after radiolabeling. Plates were coated either with TREM2 (50 μg/well) or TfR (25 μg/well) overnight. After 2 h of blocking with bovine serum albumin (BSA) (Sigma) (1 %), labeled and unlabeled mAb1729-scFv8D3_CL_ was applied to the ELISA plates in a 5 times dilution series at a starting concentration of 50 nM and incubated for 3 h. Detection was performed with secondary HRP conjugated antibodies (TfR coat: anti myc-tag, (BioNordika, Solna, Sweden); TREM2 coat: anti-his-tag (Nordic biosite, Täby, Sweden)) and incubated for 2 h. Signal was developed with K-blue Aqueous TMB substrate (LumiraDx, Solna, Sweden) and read with a spectrophotometer at 450 nm.

### Animals and *In Vivo* Experiments

*In vivo* experiments were performed in transgenic mouse models harboring the Swedish (Swe) [25] or the Swe and the Arctic mutation (ArcSwe) [25, 26] (Sup. Table 1). Prior and during the experiments, animals were housed at controlled temperature (23 °C) and humidity (50 %) at an animal facility at Uppsala University, with free access to food and water. Brain tissue from one group of previously studied ArcSwe mice that received BACE-1 inhibitor NB360 supplemented food during 3 months, starting at the age of 10 months was also included in the study [27]. The experiments were approved by The Uppsala Country Animal Ethics board (approval 5.8.18-13350/2017).

*Ex vivo* mice were i.v. administered with [^125^I]mAb1729-scFv8D3_CL_, 0.37 MBq ± 0.12 Mbq (0.3 MBq/μg). At the terminal time point, all mice were anaesthetized with 3 % isoflurane and underwent transcardiac perfusion with 40 ml 0.9 % NaCl during 2.5 min. Main organs were isolated after perfusion, and radioactivity was measured in a Wizard 2470 gamma counter (PerkinElmer). Organ concentrations were expressed as percentage of injected dose (%ID = activity (Bq/g tissue) / injected dose (Bq) ) or as standard uptake value (SUV = activity (Bq/g tissue) * body weight (g) / injected dose (Bq)).

Mice included in the PET experiment were given 0.5 % NaI in their drinking water 1 day prior to the injection of the radioligand to reduce thyroidal uptake of I-124. Mice were injected with 10.7±2.1 MBq [^124^I]mAb1729-scFv8D3_CL_ (1.3 MBq/μg). After the injection, NaI concentration in the drinking water was decreased to 0.2 % NaI. Blood samples, 8 μl, were obtained at 1 h, 3 h, 24 h, and 72 h after injection. Mice were PET scanned in pairs, one ArcSwe and one WT, at 24 h, 48 h, and 72 h after injection. Animals were anesthetized with 3 % sevoflurane or 1.8 % isoflurane and PET scanned for 60 min either in a preclinical nanoScan PET/MRI system (Mediso Medical Imaging Systems, Budapest, Hungary) followed by a CT in a preclinical SPECT/CT (Mediso) or in a Triumph Trimodality System (TriFoil Imaging Inc., Chatsworth, USA). Images from the Mediso system were reconstructed using a Tera-Tomo^TM^ 3D algorithm (Mediso) with 4 iterations and 6 subsets. Data obtained with the Triumph system were reconstructed using a 3-dimensional ordered-subsets expectations maximization with 20 iterations.

All subsequent processing of the images was performed with Amide version 1.0.4 [28]. PET and CT scans were manually aligned with a T2-weighted mouse brain atlas [29] to quantify activity in regions of interest (Supplementary Fig. 1). Brain regions used for analysis were cortex, thalamus, caudate, and hippocampus. The area under the brain concentration curve (AUC) based on the three PET scans at 24 h, 48 h, and 72 h post injection was calculated for each animal and each region of interest.

### Brain Tissue Extraction

After isolation, brains were immediately dissected in halves. The cerebellum of the left hemisphere was separated from the cerebrum, and both parts (from now on called cerebellum and brain) were directly frozen for later tissue extraction. The right hemisphere was frozen for sectioning and subsequent immunohistochemistry and autoradiography analysis. Radioactivity in the different brain tissue samples (right hemisphere, brain and cerebellum) and blood was measured. Brain and cerebellum of the left hemisphere were homogenized separately with a Precellys Evolution (VWR, Stockholm, Sweden) (4×10s at 5500 rpm) at a 1:5 weight/volume ratio in Tris-buffered saline (TBS) with complete protease inhibitor (Sigma). After centrifugation, 1 h at 16 000g, the supernatant was immediately removed and frozen.

### ELISA

Soluble Aβ protofibrils in brain TBS extracts were analyzed with a homogenous sandwich ELISA based on the Aβ N-terminal specific antibody 3D6 [30]. 96-well plates were incubated with 3D6 antibody (in house produced as described previously [22]) with 50 ng per well over night at 4 °C. Plates were blocked for 2 h with 1 % BSA. Brain extracts were incubated overnight at 4 °C followed by incubation with biotinylated 3D6 for 2 h. After 1-h incubation with streptavidin-HRP (Mabtech AB, Nacka strand, Sweden), signals were developed with K-blue Aqueous TMB substrate (LumiraDx) and read with a spectrophotometer at 450 nm.

sTREM2 levels in brain TBS extracts were analyzed using a sandwich ELISA [10]. Plates were coated with AF1729 (R&D) (20 ng per well) overnight at 4 °C followed by 2-h blocking with 1 % BSA. Brain extracts were further diluted 80 times and incubated overnight at 4 °C. Biotinylated BAF1729 (R&D) was used as a secondary antibody for 2 h followed by streptavidin-HRP conjugate (Mabtech AB) for 1 h. Signals were developed and read as above.

The Aβ and sTREM2 levels were also determined in brain extracts from all animals administered with the novel radioligand as well as in extracts obtained from a group of ArcSwe mice of different ages that were included to investigate the age or BACE-1 treatment related changes in the brain concentrations of these two proteins (Sup. Table 1).

### Autoradiography

Animals administered with similar amount of [^125^I]mAb1729-scFv8D3_CL_ were selected for autoradiography. One day after isolation of the brain, right hemispheres were cyrosectioned (40 μm) with a CM 1850 (Leica biosystems, Mölndal, Sweden). Sections were exposed to a phosphor screen in an X-ray cassette (PerkinElmer) for 16 days. The phosphor screen was read with a Cyclone Plus Imager system (PerkinElmer) at a resolution of 300 dpi. Further image processing was done with ImageJ software version 1.52a. Images were compiled with Photoshop 2020 (Adobe, San Jose, USA).

### Immunohistochemistry and Nuclear Track Emulsion

Sections from fresh frozen right hemisphere tissue were fixed for 20 min in 4 % paraformaldehyde in PBS. Antigens were retrieved by boiling the tissue for 5 s in 25 mM sodium citrate at pH 6 followed by cooling down for 20 min. Slides were washed with PBS-TritonX100 0.4 % (Sigma) for 10 min and blocked with M.O.M basic kit (BioNordika) with 5 % normal goat serum (BioNordika) for 1 h. Primary antibodies (Aβ: 6E10, Nordic Biosite; Anti-Iba-1: Wako chemicals; Täby, Sweden; Anti-GFAP: Abcam, Cambridge, UK) were applied in PBS. Antibodies were incubated over night at 4 °C on a shaker. Alexa-conjugated secondary anti-mouse, anti-rabbit, and anti-chicken antibodies (Thermo Fisher, Stockholm, Sweden) in 0.1 % Tween20 were applied for 2 h at room temperature (RT) as secondary antibodies.

### Nuclear Track Emulsion Autoradiography

Nuclear track emulsion autoradiography (NTE) was performed after immunohistochemistry according to the producer’s instructions (Ilford, Oxford, UK) as previously described [11]. In short, all experimental steps and storage were done in complete darkness. Slides were dipped in 50 % Ilford K5 emulsion in water for 5 s. Slides were dried for 2 h at RT and stored for 6 weeks at 4 °C. Slides were developed in Ilford Phenisol for 4 min at a solution temperature of 18 °C. The reaction was stopped with Ilford Ilfostop for 1 min. Slides were fixed for 4 min in Ilford Hypam and washed for 10 min in water. Slides were mounted with Biotium Everbrite mounting media (BioNordika). Pictures were taken with a Zeiss LSM700 confocal laser scanning microscope (Carl Zeiss AB, Stockholm, Sweden) and processed with ZenZeiss software (Carl Zeiss AB).

### Statistical Analysis

Data was analyzed with GraphPad Prism 6 (GraphPad Software, San Diego, USA). Groups were analyzed by non-parametric *t*-test, and multiple comparisons were performed with one-way ANOVA using Bonferroni’s *post hoc* test or Dunnet’s *post hoc* test. Average PET-based AUCs in the four regions of interests were ranked and compared using a non-parametric Mann-Whitney test. Results are reported as averages and standard deviation: *p < 0.05, **p < 0.01, ***p < 0.001, and ****p <0.0001.

## Results

### TREM2 Levels Increase with Age

**Fig. 2:**
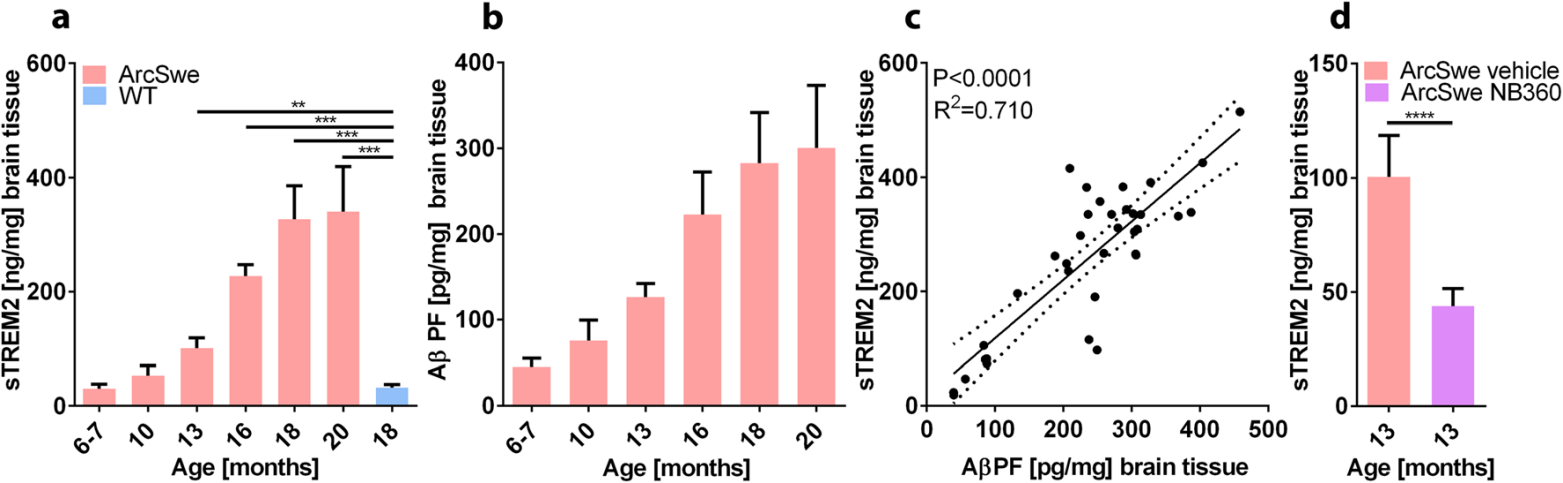
Analysis of homogenized post mortem brain tissue obtained from ArcSwe mice of different ages. (**a**) sTREM2 concentrations in TBS brain extracts in age groups 6–7 months (*n*=7), 10 months (*n*=8), 13 months (*n*=8), 16 months (*n*=5), 18 months (*n*=11), 20 months (*n*=14), and wild-type (WT) 18 months old (*n*=18). (**b**) Aβ protofibril (PF) concentrations in TBS brain extracts in the same age groups (except WT as they do not express human Aβ). (**c**) Correlation between sTREM2 and Aβ protofibril concentration. (**d**) Decreased sTREM2 levels in mice treated with BACE-1 inhibitor NB360 (*n*=9) compared to vehicle treated animals (*n*=8) at the age of 13 months.

sTREM2 and Aβ protofibril concentrations were determined in the TBS soluble fraction of homogenates prepared from brains isolated from ArcSwe mice of different ages: 6–7, 10, 13, 16, 18, and 20 months (Fig. 2a–b). sTREM2 levels increased with age and correlated closely with Aβ protofibrils (Fig. 2c). A large increase of both of these proteins was observed between the age of 13 and 18 months, while the concentrations then appeared to level out between 18 and 20 months. Brain tissue isolated from ArcSwe mice that had received the anti-Aβ drug NB360 [31] (BACE-1 inhibitor) with confirmed reduction of Aβ pathology [27] displayed reduced sTREM2 brain concentrations compared to littermates that did not receive NB360. This provided further mechanistic evidence of the strong correlation between Aβ and sTREM2 (Fig. 2d).

### Generation of a Bispecific Antibody-Based Radioligand for TREM2

**Fig. 3:**
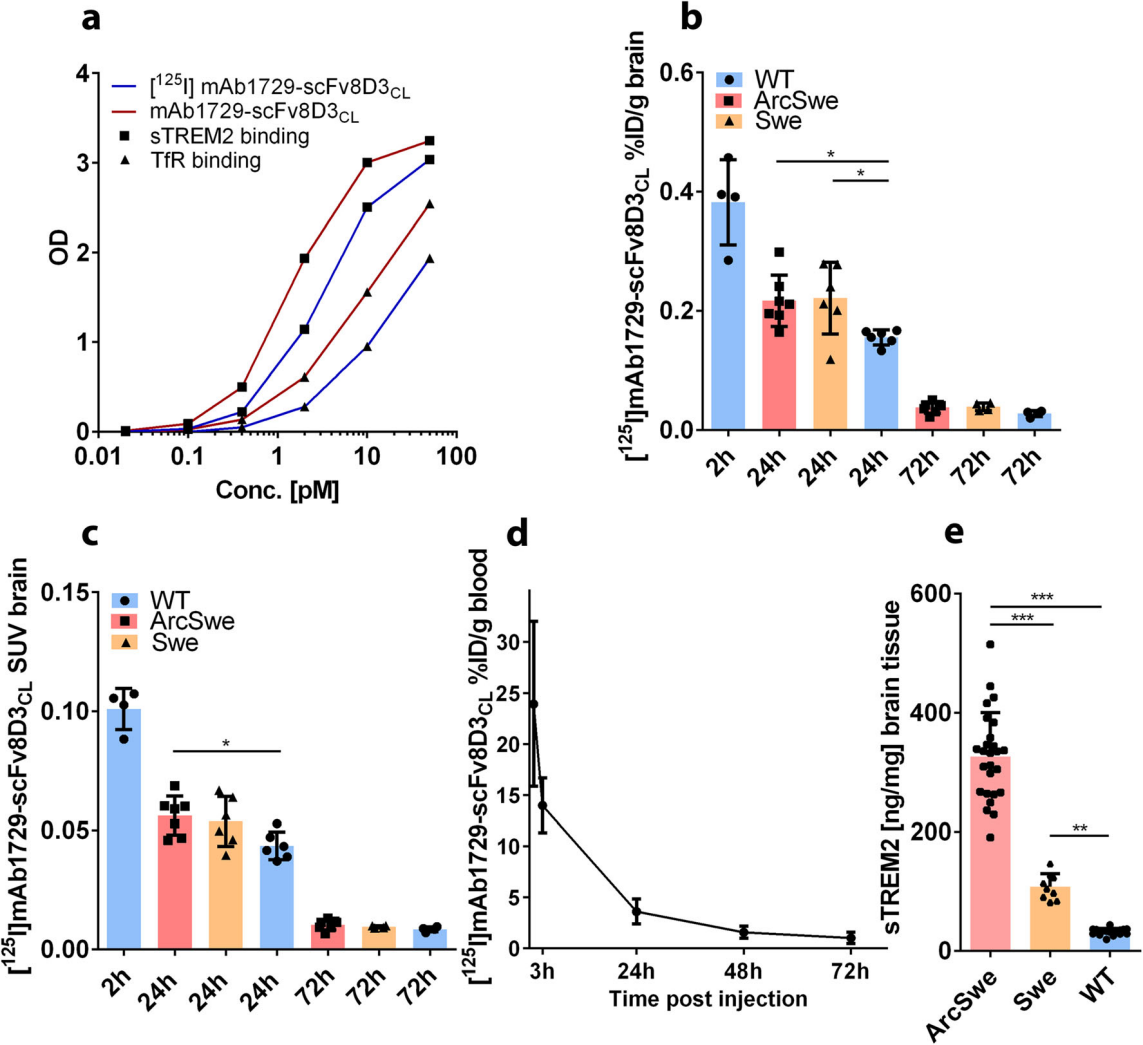
(**a**) Binding comparison of I-125 labeled and unlabeled mAb1729-scFv8D3_CL_ in ELISA. (**b**) Percent of injected dose and standard uptake value (SUV) (**c**) of [^125^I]mAb1729-scFv8D3_CL_ in brain 2 h, 24 h, and 72 h after injection. (**d**) Blood was sampled 1 h, 3 h, 24 h, 48 h, and 72 h after injection. (**e**) sTREM2 levels in TBS extracted brains of ArcSwe, Swe, and WT mice at the age of 18 months.

Anti-TREM2 antibody mAb1729 was linked to the TfR binding scFv8D3_CL_ by chemical conjugation between a TCO group attached to mAb1729 and a tetrazine group attached to scFv8D3_CL_. After fusion, both binding moieties, i.e., scFv8D3_CL_ and mAb1729, remained functional and bound to their targets, i.e., TfR and TREM2, respectively (Fig. 3a). The bispecific construct was then radiolabeled with I-125, and functionality of the radioligand was investigated both *in vitro* and *in vivo*. The binding affinity to both TfR and TREM2 was decreased somewhat after radiolabeling (Fig. 3a). However, when administered to WT mice, the brain concentrations at 2 h were around 0.4 % of injected dose per gram brain tissue (%ID/g) (Fig. 3b) which is in line with what has been reported for similar constructs previously [11] and thus indicated that the scFv8D3_CL_ binding moiety remained functional also after radiolabeling.

To study TREM2 levels *in vivo*, [^125^I]mAb1729-scFv8D3_CL_ was administered intravenously to 18–20-month-old transgenic ArcSwe, Swe, and WT mice. Following administration, the radioligand was cleared from the blood with an estimated half-life of about 13 h (Fig 3d, Supplementary Fig. 2a). Brain concentration of [^125^I]mAb1729-scFv8D3_CL_ in isolated brains was measured at 24 h and 72 h post injection and expressed as %ID/g (Fig. 3b) or as SUV (Fig. 3c). At 24 h post injection, [^125^I]mAb1729-scFv8D3_CL_ brain concentrations were 29 % higher in ArcSwe animals (*p*=0.042) and 30 % higher in Swe animals (*p*=0.036) compared to WT, when expressed as %ID/g. When expressed as SUV, brain concentrations were 23 % higher in ArcSwe (*p*=0.029) and 19 % higher in Swe mice (*p*=0.049) compared to WT. At 72 h post injection, neither of the transgenic models showed significantly different [^125^I]mAb1729-scFv8D3_CL_ concentrations (*p*>0.05) compared to WT. However, there was a trend that the transgenic animals also at this time point showed elevated [^125^I]mAb1729-scFv8D3_CL_ brain concentrations compared to WT (Fig. 3b–c). sTREM2 concentrations determined in brain homogenates with ELISA were elevated in ArcSwe mice in comparison to both Swe and WT (Fig. 3e). Swe mice also displayed higher sTREM2 brain concentrations than WT mice (Fig. 3e).

### *In Vivo* PET and *Ex Vivo* Autoradiography Imaging of TREM2

**Fig. 4:**
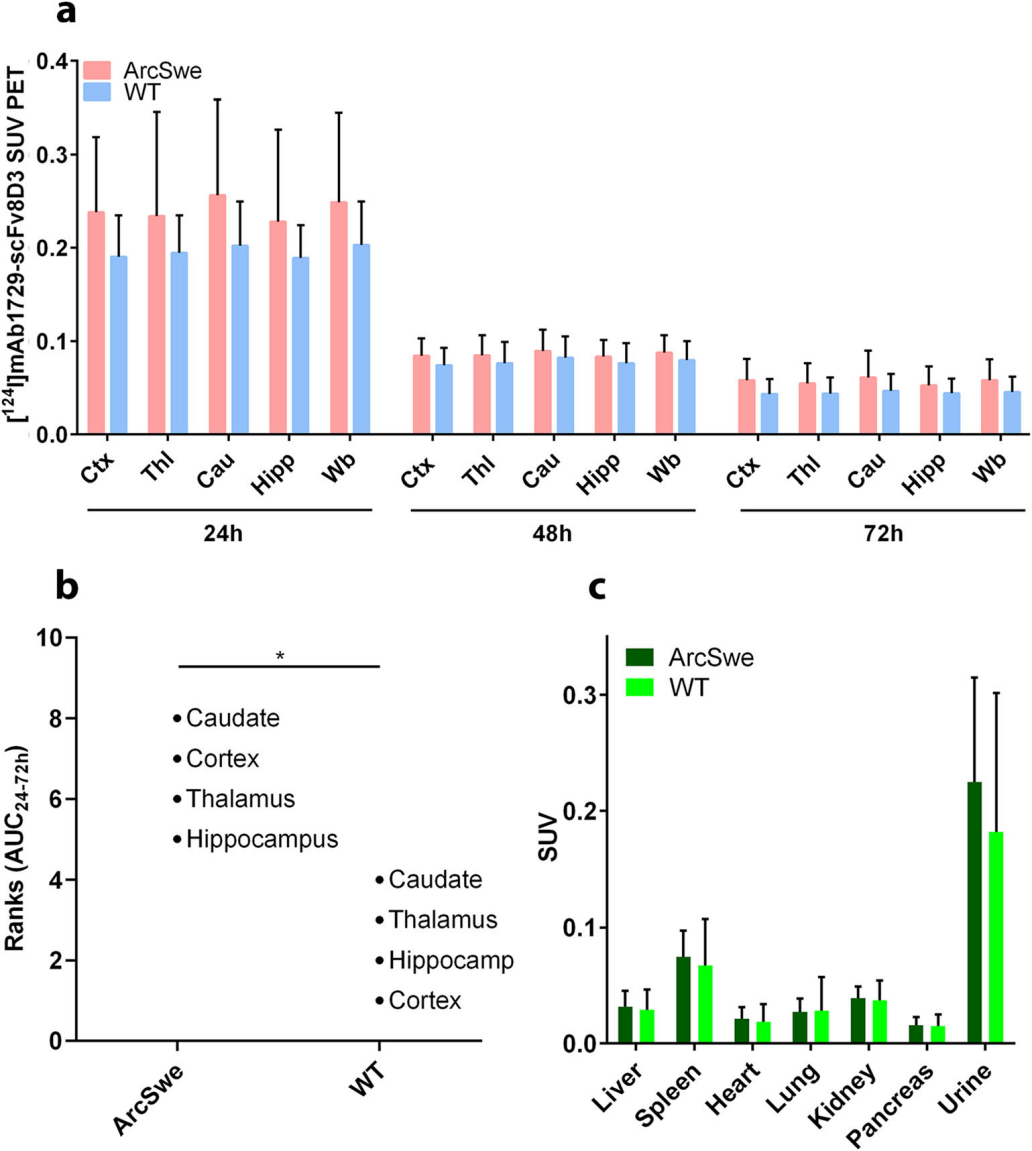
(**a**) Quantification, expressed as SUV, of the radioligand [^124^I]mAb1729-scFv8D3_CL_ in cortex (Ctx), thalamus (Thl), caudate (Cau), hippocampus (Hipp), and the whole brain (Wb) with PET at 24 h, 48 h, and 72 h after injection in ArcSwe (*n*=8) and WT (*n*=8). (**b**) Ranks of AUC, calculated between 24 h and 72 h. Caudate in ArcSwe displayed the highest exposure of the radioligand, while cortex in WT showed the lowest exposure. (**c**) Biodistribution of [^124^I]mAb1729-scFv8D3_CL_ in organs and urine of ArcSwe and WT mice. No difference was observed in the peripheral distribution between ArcSwe and WT.

ArcSwe and WT mice were scanned at 24 h, 48 h, and 72 h post [^124^I]mAb1729-scFv8D3_CL_ injection. Brain concentrations of [^124^I]mAb1729-scFv8D3_CL_ were quantified as SUV in cortex, thalamus, caudate, hippocampus, and the whole brain (Fig. 4a). Although not reaching statistical significance, ArcSwe mice tended to display higher SUVs at all time points in all investigated regions. To estimate the brain exposure over time, the area under the concentration curve (AUC_24-72 h_) was calculated for all sub-regions. When ranked, AUCs in all four investigated sub-regions in ArcSwe mice were larger than the AUCs calculated in the WT mice (Fig. 4b, Supplementary Fig. 2b).

**Fig. 5:**
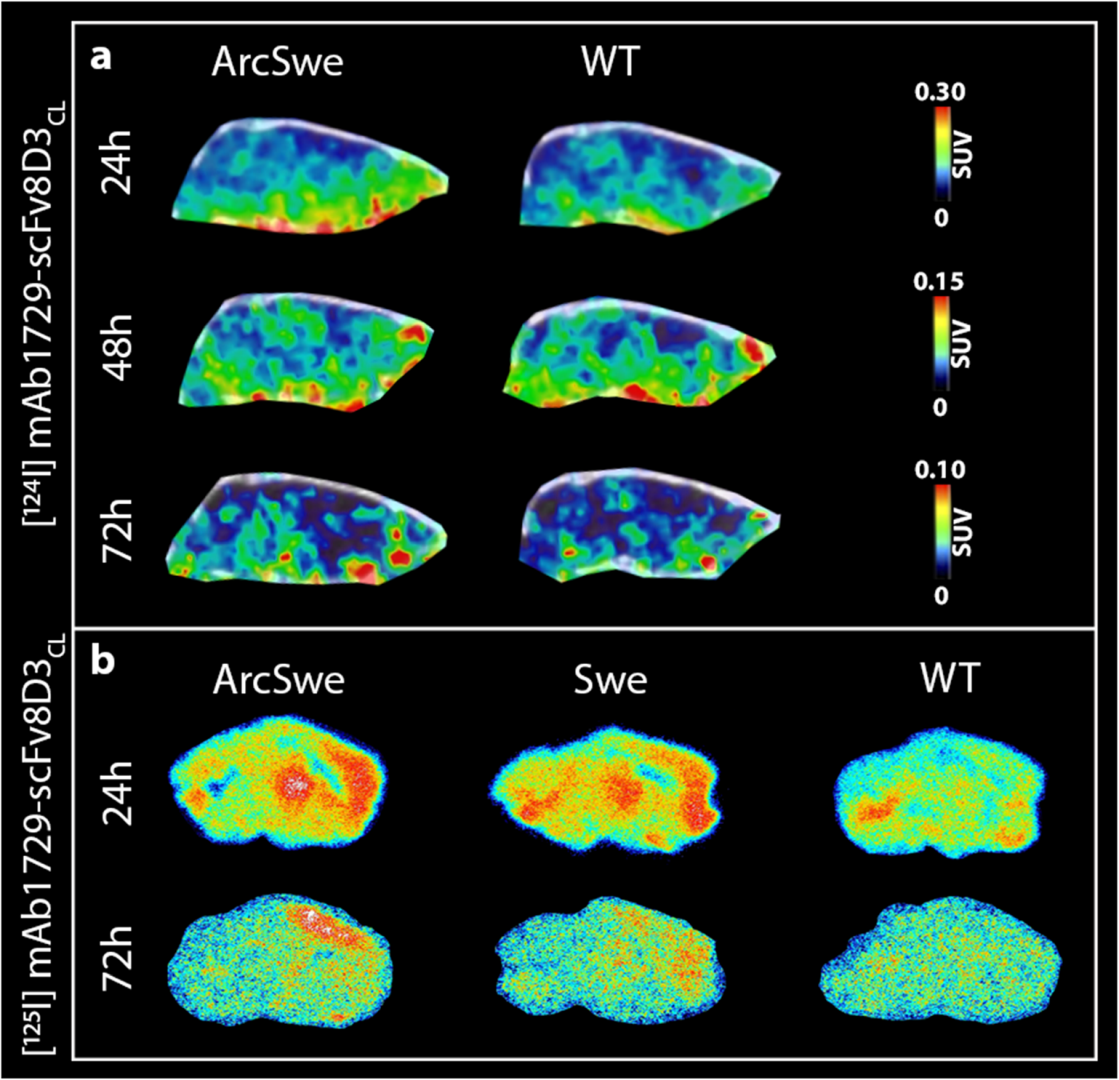
Representative SUV scaled sagittal PET images with [^124^I] mAb1729-scFv8D3_CL_. ArcSwe animals, compared to WT animals at 24 h, 48 h, and 72 h after injection (**a**). Radioligand distribution in brain tissue displayed in sagittal *ex vivo* autoradiography images in ArcSwe, Swe, and WT animals at 24 h and 72 h after injection (**b**).

Radioligand concentrations in liver, spleen, heart, lung, kidney, pancreas, and urine after the 72 h scan were quantified *ex vivo* after perfusion. The concentration of [^124^I]mAb1729-scFv8D3_CL_ in these organs were not significantly different between ArcSwe and WT mice (Fig. 4c, Supplementary Fig. 2c). In line with the single time point brain SUVs, PET images did not reveal any visual difference between ArcSwe and WT mice (Fig. 5a).

Autoradiography was performed on brain sections prepared from the right hemisphere of the perfused brains to study the spatial distribution of [^125^I]mAb1729-scFv8D3_CL_ within the brain tissue without radioactivity from the brain blood volume (which is included in the signal *in vivo* with PET). The intensity of the signal appeared to be higher in ArcSwe and Swe compared with WT animals in sections obtained from animals perfused at 24 h (Fig. 5b and Supplementary Fig. 3). In particular, ArcSwe and Swe animals displayed increased retention of [^125^I]mAb1729-scFv8D3_CL_ in cortex and thalamus, two regions known to contain high levels of Aβ, compared to other regions. Although the signal at 72 h was low, there was still a visually detected increased signal in cortex of ArcSwe animals.

### Immunohistochemistry and Nuclear Track Emulsion (NTE)

**Fig. 6:**
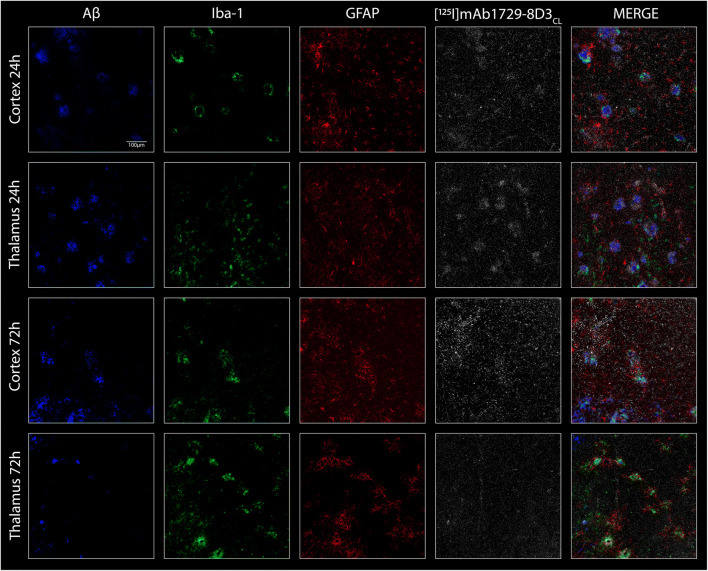
Immunohistochemistry and nuclear track emulsion. Tissue was stained for Aβ, Iba-1, and GFAP on sections prepared from perfused and snap-frozen ArcSwe brains 24 h and 72 h post injection of [^125^I]mAb1729-scFv8D3_CL_. Nuclear track emulsion visualizes the retention of [^125^I]mAb1729-scFv8D3_CL_ in the tissue, white dots represent radiolabeled antibody.

Brains sections from ArcSwe animals were stained for Iba-1, GFAP, and Aβ to map activated microglia and astrocytes in connection with Aβ plaque pathology (Fig. 6, Supplementary Fig. 4). NTE was performed on the stained sections in order to visualize the retention of [^125^I]mAb1729-scFv8D3_CL_ in the tissue. Cortex and thalamus were chosen because of high Aβ load and high retention of the radioligand on autoradiography images. At 24 h after injections, [^125^I]mAb1729-scFv8D3_CL_ was found in the vicinity of Aβ plaques and activated glial cells. However, the antibody did not appear to co-localize with astrocytes and only partly with microglia. At 72 h after injection, the radioligand was more equally distributed throughout the whole tissue, and there was no longer a pattern of high concentration of [^125^I]mAb1729-scFv8D3_CL_ in association to Aβ plaques.

## Discussion

In this study, we designed and generated a bispecific, brain-penetrant antibody and used it to assess TREM2 levels in two different mouse models of Aβ pathology.

Since the first investigations two decades ago, upregulated TSPO levels have been the center of attention for studies of microglial activation *in vivo* with PET. However, the development of radioligands for activated microglia has turned out to be challenging. The first and most frequently used PET radioligand [^11^C]PK11195 is associated with problems such as high plasma protein binding, low extraction fraction to the brain, and limited specific-to-background ratios [32, 33]. The use of later generations of TSPO PET radioligands is hampered by a nucleotide polymorphism in the TSPO gene that affects binding [34]. Third-generation radioligands, such as [^18^F]GE180, are currently under intense research and seem, in line with the second generation of radioligands, to be affected by polymorphisms although to a lesser degree. Similar to [^11^C]PK11195, low first-pass extraction rate and a large blood component have been observed also for the more recently developed TSPO radioligands [35]. Further, and regardless of radioligand generation, TSPO is not specific for microglia but also expressed on astrocytes and endothelial cell as well as on peripheral cells of myeloid lineage [36, 37]. Thus, alternatives to TSPO for imaging of microglial activation are needed. One such alternative could be imaging if TREM2. The radioligand [^125^I]mAb1729-scFv8D3_CL_, described in the present study, enters the brain by TfR mediated transcytosis across the BBB. The brain concentrations at 1 day after administration were comparable to what has been described previously for bispecific constructs based on TfR and Aβ targeting antibodies [11]. Similar to the bispecific radioligands for different Aβ species, the new radioligand for TREM2, [^125^I]mAb1729-scFv8D3_CL_, showed increased brain retention in transgenic mice compared to wild-type mice at 1 day post injection. These results were in line with sTREM2 levels measured in *post mortem* brain tissue by ELISA.

However, with PET, the study failed to clearly visualize and quantify differences between transgenic and WT mice *in vivo* at a single time point (24 h, 48 h, or 72 h), despite the convincing differences in sTREM2 brain concentrations. One difference between *in vivo* PET and *ex vivo* ELISA detection is, as discussed previously, the presence of membrane bound TREM2 *in vivo*. Thus, in theory, the methods may detect slightly different “pools” of TREM2. Still, autoradiography as well as NTE indicated that the radioligand was present at higher levels in mice with Aβ pathology compared to WT. These observations were also supported by the AUC comparisons between transgenic and WT mice based on PET measurement on multiple days (24–72 h). One obvious challenge with antibodies as PET radioligands is their long residence time in blood. Since 3 % of the brain volume is blood [38], a high signal in blood will mask the signal from the brain parenchyma. In the present study, when blood was removed, as in *ex vivo* autoradiography, differences between transgenic and WT animals became visible already at 24 h post injection. Thus, a facilitated clearance could enable *in vivo* visualization at this time point. Reducing the size of the bispecific construct has previously been shown to increase the rate of elimination [14]. Scanning at a later time point, when blood concentrations have decreased, is also a possibility if the radioligand is retained at its intrabrain target. In the present study, however, it appeared that the radioligand, or potentially the radioligand bound to sTREM2, was not retained in the brain over a longer period of time. Radioligand concentrations decreased from 0.38 %ID/g at 2 h to 0.21 %ID/g already at 24 h post injection. Compared to previous bispecific constructs targeting Aβ, the initial concentrations are similar, but for Aβ directed constructs, concentrations as low as 0.2 %ID/g are not reached until 72 h post injection in transgenic mice [11]. One possible explanation for this striking difference may be the nature of the target. sTREM2 is soluble and highly diffusible and might be removed from the brain at a higher rate than large Aβ aggregates. The binding of mAb1729-scFv8D3_CL_ to sTREM2 or potentially to membrane bound TREM2 could facilitate the clearance. NTE also showed that less mAb1729-scFv8D3_CL_ appeared to be localized in vicinity of the plaques at a later time point. Yet another possibility is that the binding affinity of mAb1729-scFv8D3_CL_ to TREM2 or sTREM2 is not sufficient to retain the antibody. Thereby, in line with the process in the WT brain, unbound mAb1729-scFv8D3_CL_ is cleared from the brain parenchyma. Thus, antibodies with higher affinity for TREM2 could result in improved PET quantification. As TREM2 is a new disease target, the availability of antibodies was limited at the start of the present study, but new antibodies are appearing [39].

In conclusion, [^125^I]mAb1729-scFv8D3_CL_ was shown to be a useful tool to detect TREM2 *ex vivo*. The study showed that antibody-based PET imaging of TREM2 is a possibility, but antibody formats that are cleared faster from blood and antibodies showing higher affinity for TREM2 must be developed to further progress this technique for *in vivo* use.

## Supplementary Information

ESM 1 (DOCX 4638 kb)
