# Supplementary material for: Pinpointing Brain TREM2 Levels in Two Mouse Models of Alzheimer’s Disease
Source: Mol Imaging Biol. 2021 Feb 23;23(5):665–75. doi: 10.1007/s11307-021-01591-3 (PMC8410720; doi:10.1007/s11307-021-01591-3)
Supplement: Supplementary file 1 — (DOCX 4638 kb) [file 11307_2021_1591_MOESM1_ESM.docx]

### **Supplementary**


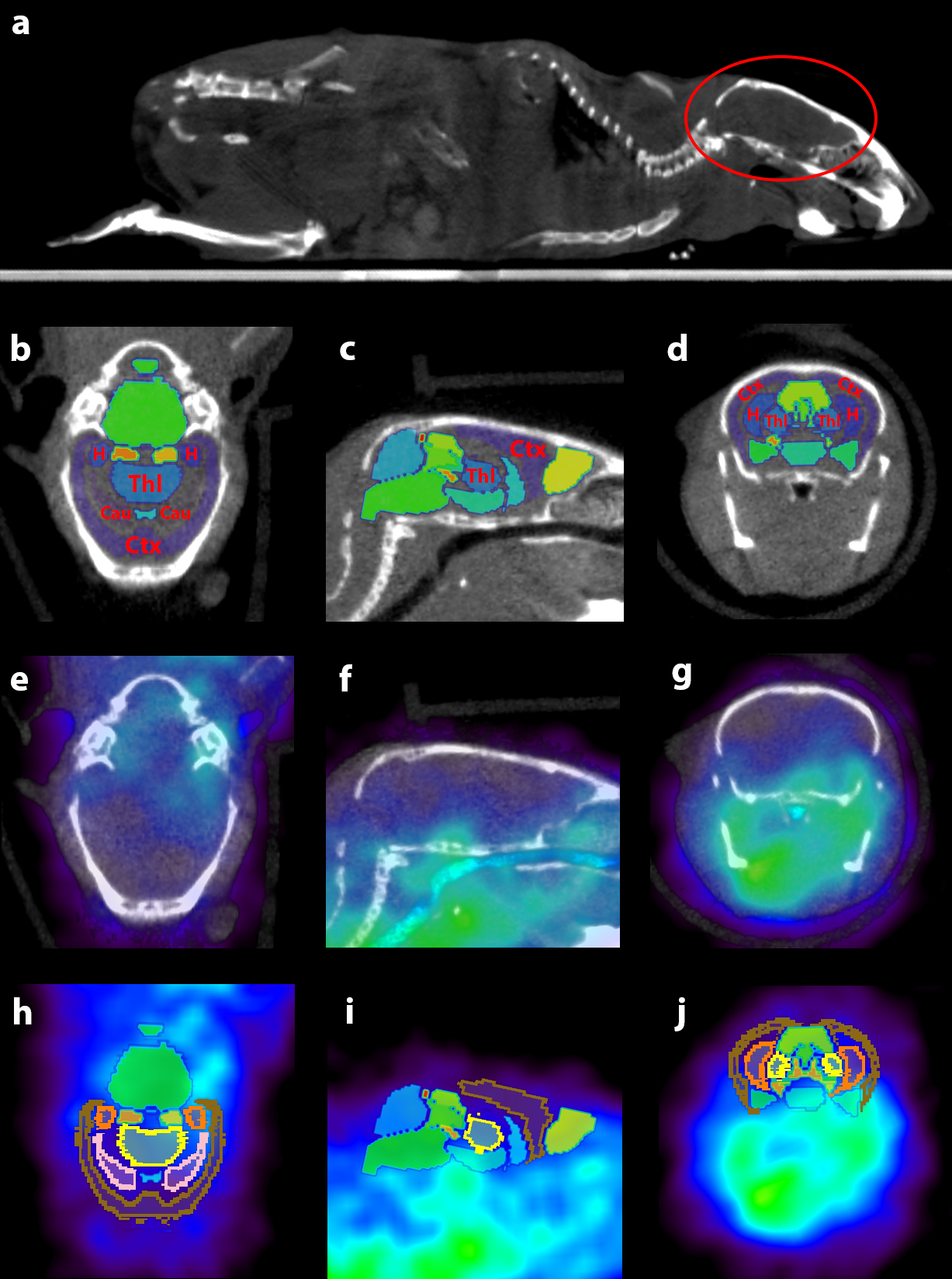


**Fig.1:** Illustration of PET data extraction **a:** whole body mouse CT. Red ellipse marks the brain region, where the brain atlas and ROIs were fitted. **b-d**: CT image with fitted ROI atals in transversal (**b**) sagittal (**c**) and coronal (**d**) plane. ROIs used in this study, e.g.cortex (Ctx), thalamus (Thl), caudate (Cau) and hippocampus (H) are marked. **e-g**: Illustration of the aligned PET image to the CT in all three planes. **h-j**: PET image with the aligned ROIs for data extraction. Contours of applied ROIs are highlighted for better contrast and visibility.

| Brain region | ArcSwe | WT |
| --- | --- | --- |
| Ctx | 5.6 ± 1.1 | 4.6 ±0.6 |
| Thl | 5.5 ± 1.4 | 4.7 ± 0.7 |
| Hipp | 5.4 ± 1.2 | 4.6 ± 0.6 |
| Cau | 6.0 ± 1.3 | 5.0 ± 0.7 |
| WB | 5.8 ± 1.2 | 4.9 ± 0.7 |


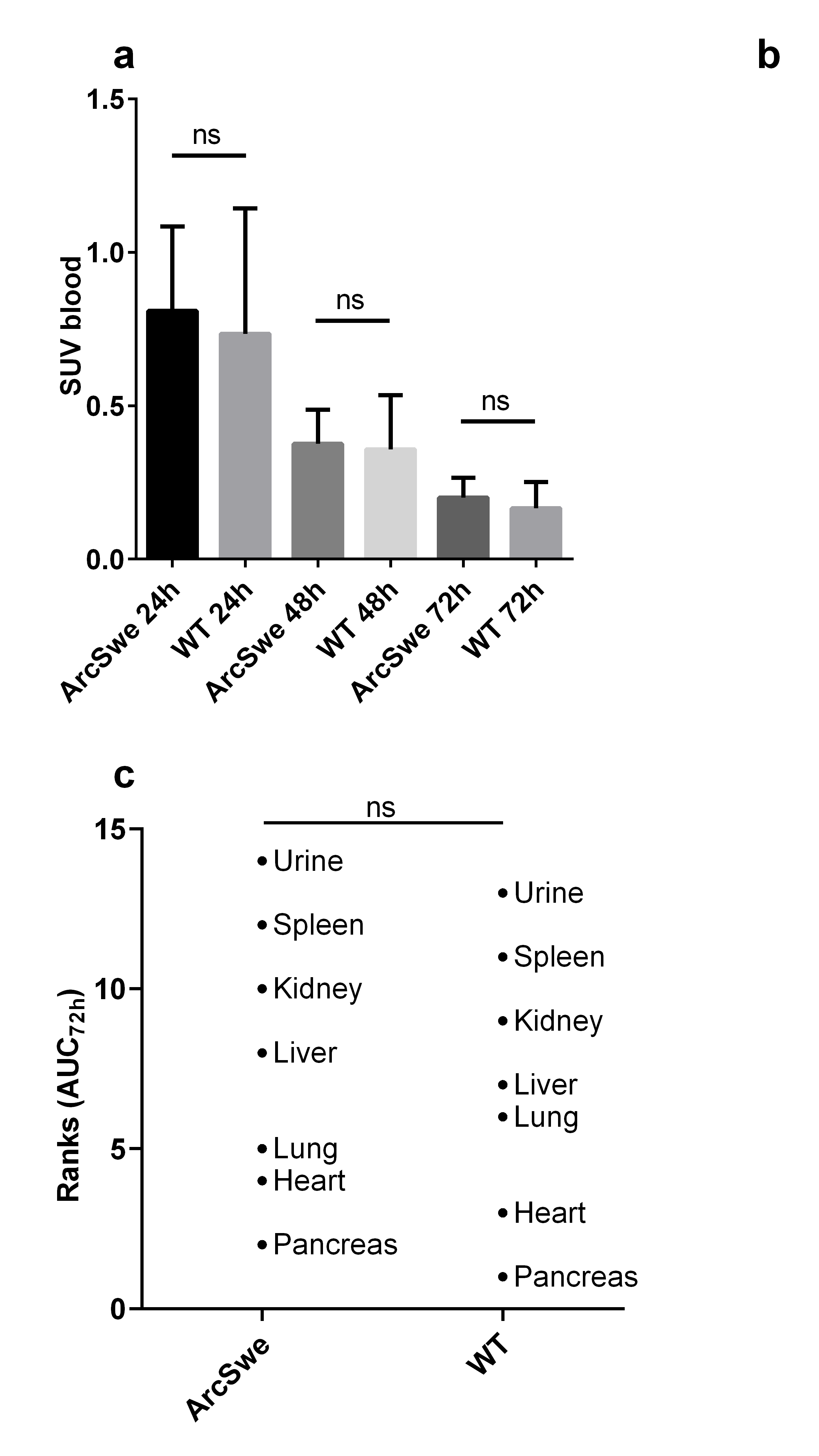


**Fig.2a**: SUV blood in transgenic and WT animals scanned with [^124^I]mAb1729-scFv8D3_CL._ Samples were taken 24h, 48h and 72h after injection. **b**: Brain region area under the curve (AUC) based on the SUV obtained from three scans acquired at 24h, 48h, and 72h with [^124^I]mAb1729-scFv8D3_CL_ (AUC± SEM). **c**: Ranks of SUV in peripheral organs at 72h after injection of [^124^I]mAb1729-scFv8D3_CL_.


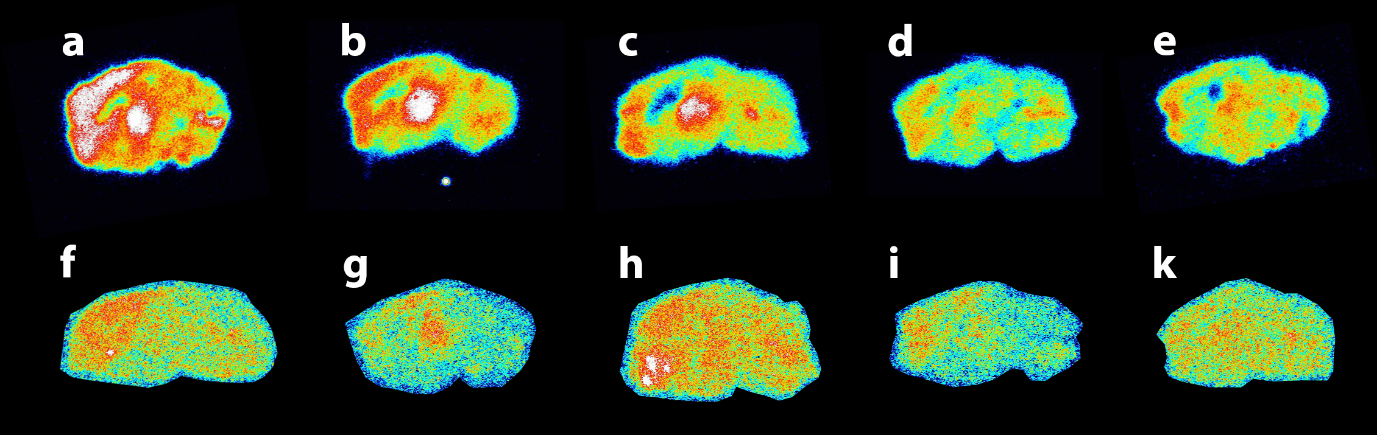


**Fig.3**: Additional autoradiography pictures of [^125^I]mAb1729-scFv8D3_CL_. (**a-c)**: ArcSwe 24 h post injection. **(d)**: Swe 24 h post injection. (**e**): WT 24 h post injection. (**f-g**): ArcSwe 72 h post injection. (**h-i**): Swe 72 h post injection. (**k**): WT 72 h post injection. NB! Animals received somewhat different amount of radioactivity and thus, images should be analyzed for differences in regional distribution rather than total intensity. For example, ArcSwe mice show high intensity signals in cortex and thalamus while this regional distinction is not observed in WT.


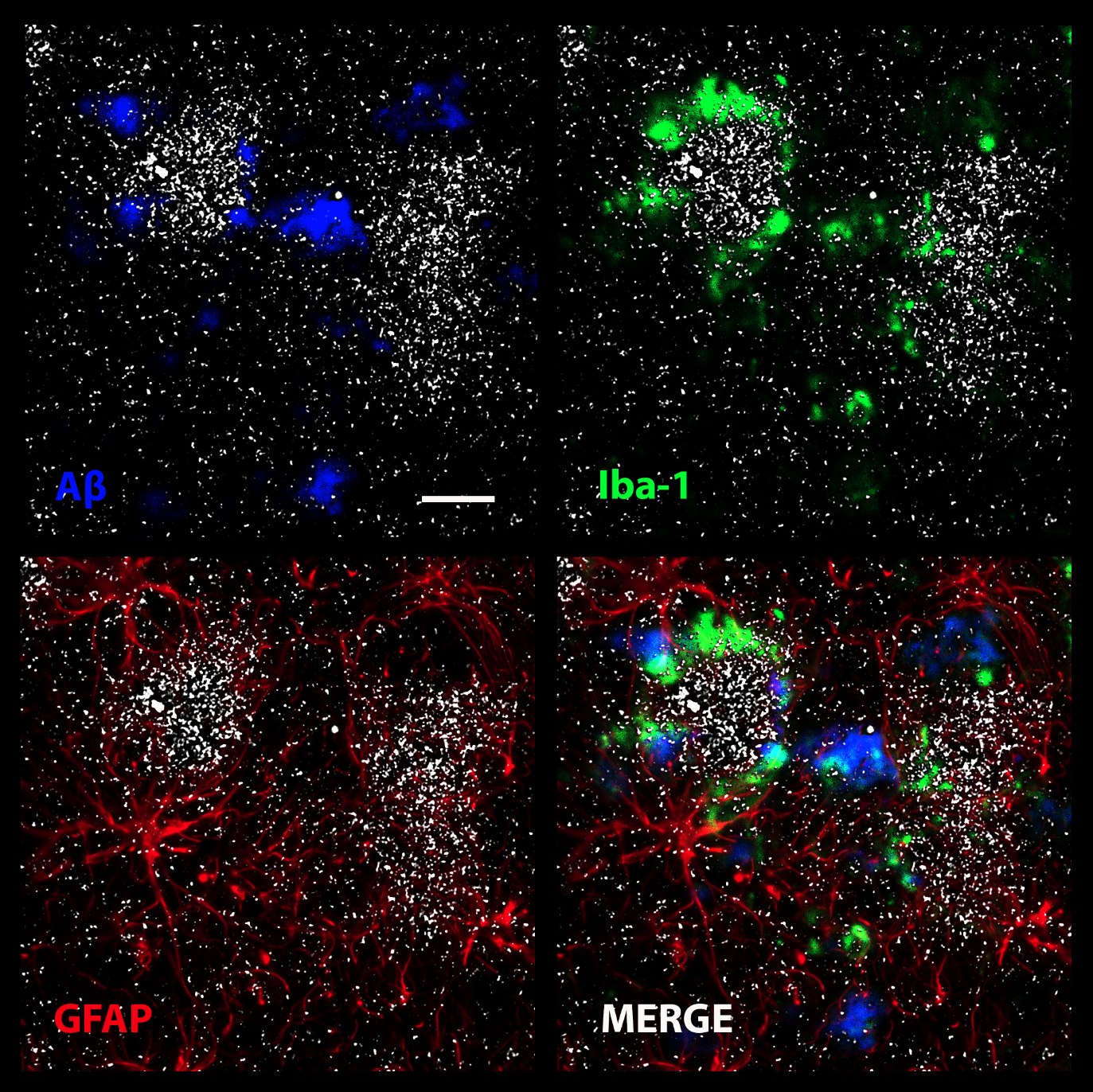


**Fig.4**: Immunohistochemistry and nuclear track emulsion in ArcSwe thalamus 24h post injection. Snap-frozen tissue was stained for Aβ (blue channel, Iba-1 (green channel) and GFAP (red channel). Nuclear track emulsion visualizes the retention of [^125^I]mAb1729-scFv8D3_CL_ in the tissue (white dots). Scale bar: 20µm.

**Table1:** Animals used in the experiments.

| **Experiment** | **Model** | **Tracer** | **Perfusion post injection** | **Age (months)** | **Number of animals (n)** |
| --- | --- | --- | --- | --- | --- |
| *Ex vivo analysis* | Swe | [^125^I]mAb1729-scFv8D3_CL_ | 24 h | 18 | 6 |
|  | ArcSwe | [^125^I]mAb1729-scFv8D3_CL_ | 24 h | 18 | 8 |
|  | WT | [^125^I]mAb1729-scFv8D3_CL_ | 24 h | 18 | 6 |
|  | WT | [^125^I]mAb1729-scFv8D3_CL_ | 2 h | 3 and 18 | 4 |
| *Ex vivo analysis* | Swe | [^125^I]mAb1729-scFv8D3_CL_ | 72 h | 18 | 5 |
|  | ArcSwe | [^125^I]mAb1729-scFv8D3_CL_ | 72 h | 18 | 7 |
|  | Wt | [^125^I]mAb1729-scFv8D3_CL_ | 72 h | 18 | 6 |
| *PET* | ArcSwe | [^124^I]mAb1729-scFv8D3_CL_ | 72 h | 18-20 | 8 |
|  | WT | [^124^I]mAb1729-scFv8D3_CL_ | 72 h | 18 | 8 |
| *sTREM2 ELISA* | ArcSwe |  |  | 6-7 | 7 |
|  | ArcSwe |  |  | 10 | 7 |
|  | ArcSwe |  |  | 13 | 9 |
|  | ArcSwe |  |  | 16 | 5 |
|  | ArcSwe |  |  | 18 | 11 |
|  | ArcSwe |  |  | 20 | 14 |
|  | WT |  |  | 18-20 | 1 |
|  | ArcSwe | NB-360 treated^27^ |  | 13 | 8 |
| *Aβ PF ELISA* | ArcSwe |  |  | 6-7 | 3 |
|  | ArcSwe |  |  | 10 | 6 |
|  | ArcSwe |  |  | 13 | 4 |
|  | ArcSwe |  |  | 16 | 8 |
|  | ArcSwe |  |  | 18 | 8 |
|  | ArcSwe |  |  | 20 | 16 |
|  | Swe |  |  | 18 | 9 |
